# The Role of Inflammatory Cytokines in the Causal Pathway From Gut Microbiota to Sjögren's Syndrome: Evidence From Mendelian Randomization

**DOI:** 10.1155/mi/1951493

**Published:** 2025-12-01

**Authors:** Junkang Zhao, Jiannan Han, Xiuying Fan, Jie Kang, Ruonan Wu, Yixuan Zhao, Lina Bai, Xue Gao, Dan Ma, Liyun Zhang

**Affiliations:** ^1^Third Hospital of Shanxi Medical University, Shanxi Bethune Hospital, Shanxi Academy of Medical Sciences, Tongji Shanxi Hospital, Taiyuan 030032, Shanxi, China; ^2^Shanxi Academy of Advanced Research and Innovation (SAARI), No.7, Xinhua Road, Xiaodian District, Taiyuan, Shanxi, China; ^3^Shanxi Province Clinical Research Center for Dermatologic and Immunologic Diseases (Rheumatic Diseases), Taiyuan, Shanxi, China; ^4^School of Public Health, Shanxi Medical University, Taiyuan 030001, Shanxi, China; ^5^Dongzhimen Hospital, Beijing University of Chinese Medicine, Beijing 100700, China; ^6^Department of Health Statistics, School of Public Health, Shanxi Medical University, Jinzhong 030606, China

**Keywords:** gut microbiota, inflammatory cytokines, mediation analysis, Mendelian randomization, Sjögren's syndrome

## Abstract

**Purpose:**

Evidence is accumulating that links gut microbiota, a crucial component of the immune environment, to Sjogren's syndrome (SS). The mechanisms underlying the influence of gut microbiota on the onset and development of SS are still not completely understood. To this end, we applied a Mendelian randomization (MR) framework to investigate whether inflammatory cytokines mediate the association of gut microbiota with SS.

**Methods:**

Our MR analysis leveraged publicly available GWAS data, including information on 211 gut microbiota taxa sourced from the MiBioGen consortium (18,340 participants), summary statistics for 91 inflammatory cytokines obtained from a study of 14,824 individuals, and genetic data for SS derived from the UK Biobank (407,746 participants). To investigate causal associations between gut microbiota and SS, we primarily employed the inverse variance weighted method, supported by additional techniques such as MR-Egger, simple mode, weighted median, and weighted mode for validation. The potential mediating effect of inflammatory cytokines in the gut microbiota–SS relationship was investigated using both mediation MR and multivariable MR (MVMR) analyses.

**Results:**

MR analysis identified five microbiota taxa causally associated with SS. Particularly, class Gammaproteobacteria (OR = 3.468, 95% CI = 1.139–10.557, *p*=0.029) and genus Peptococcus (OR = 1.722, 95% CI = 1.082–2.471, *p*=0.022) were significantly associated with an increased risk of developing SS. Six inflammatory cytokines were identified as potentially causal, with Axin-1 (OR = 2.556, 95% CI = 1.072–6.096, *p*=0.034) and C-X-C motif chemokine 10 levels (CXCL10) (OR = 3.049, 95% CI = 1.428–6.513, *p*=0.004) being the most critical contributors. Mediation MR analysis showed that Axin-1 levels mediated 16.96% of the causal effect of class Gammaproteobacteria on SS, CXCL10 levels mediated 36.78% of the causal effect of genus Coprococcus3 on SS.

**Conclusion:**

The findings suggest that certain gut microbiota is sociated with an increased risk of SS, mediated by specific inflammatory cytokines.

## 1. Introduction

Sjögren's syndrome (SS) is a systemic autoimmune condition that predominantly occurs in women after their fourth decade of life. Its hallmark clinical manifestations—ocular and oral dryness—stem from immune-mediated inflammation targeting the lacrimal and salivary glands [1]. The prevalence of SS in the Chinese population is between 0.33% and 0.77%, making it the most common autoimmune connective tissue disease among middle-aged and elderly individuals [2]. Due to abnormal T-cell and B-cell responses to self-antigens—specifically the ribonucleoprotein particles Ro/SSA and La/SSB expressed by epithelial cells in salivary and lacrimal glands—cytokine and chemokine levels become elevated, triggering chronic inflammation in exocrine glands that ultimately leads to loss of physiological function [3]. Reduced secretion from lacrimal and salivary glands affects patients systemically, with ~30% developing multi-organ involvement, 5% progressing to malignant lymphoma [4], and others experiencing severe organ-specific manifestations such as interstitial lung disease [5] and renal failure [6], alongside increased risks of opportunistic infections and cardiovascular disease due to immunosuppression [7]. The gut microbiome is increasingly recognized as a fundamental modulator of the immune system, with studies demonstrating its profound influence on immune cell dynamics [8]. Dysbiosis of the gut microbiota has been associated with a higher susceptibility to autoimmune diseases by disrupting immune homeostasis [9]. Given the limited effective treatments available, the substantial burden of SS underscores the critical need for comprehensive studies into its etiology. Understanding the underlying causes of SS is essential for developing therapeutic strategies that are effective and minimally invasive.

Although the exact causes and mechanisms underlying SS are not fully understood, a combination of genetic factors, environmental influences, and their interactions play a significant role in its development. Moreover, emerging evidence suggests that disturbances in gut microbiota can compromise intestinal mucosal barriers, disrupt immune function, elevate inflammatory markers, and contribute to chronic inflammation, thereby influencing the onset and progression of SS [10, 11]. The gut microbiota, comprising the largest commensal microbial community in the human body, resides within the gastrointestinal tract. It plays a crucial role in shaping the development and function of the human immune system, which may affect susceptibility to autoimmune diseases. Additionally, the gut microbiota serves to inhibit the proliferation of pathogenic organisms, maintaining a balanced microbial environment essential for overall health [12].

In addition, cytokines have become a key factor in the current debate on the etiology of the disease [13]. The gut microbiota is known to induce an inflammatory response by promoting the differentiation and recruitment of Th1, Th17, and CD8+ T cells, while simultaneously stimulating the expression of pro-inflammatory mediators, including IFN-γ, IL-1β, IL-6, TNF-α, and IL-17. These cytokines are known to exacerbate inflammation and contribute to the destruction of the lacrimal gland, which is a characteristic feature of SS [14, 15]. Inflammatory cytokines are likely key mediators in this pathway [16, 17]. Additionally, patients with SS, particularly primary SS (pSS), often exhibit reduced levels of butyrate, a short-chain fatty acid (SCFA) produced by gut bacteria. Butyrate plays a crucial role in supporting a diverse bacterial flora in the gut. Its deficiency can compromise intestinal barrier function, leading to increased permeability and allowing for the translocation of bacteria and microbial products. This breach in the intestinal barrier can trigger immune responses characterized by elevated production of pro-inflammatory cytokines, while simultaneously reducing the release of anti-inflammatory cytokines. These imbalances contribute to chronic inflammation observed in patients with SS, thereby exacerbating the autoimmune response [18]. Based on this evidence, a causal pathway linking gut microbiota, inflammatory cytokines, and SS appears plausible. This study seeks to elucidate these relationships and pinpoint specific inflammatory cytokines with potential applications in early diagnosis and clinical management of SS.

Mendelian randomization (MR) employs genetic variants as instrumental variables (IVs) to infer causal effects between exposures and outcomes, thereby mitigating confounding influences and minimizing reverse causation bias [19]. In light of this evidence, we implemented a bidirectional two-sample MR framework combined with mediation analysis, using publicly available GWAS summary data to investigate the role of inflammatory cytokines in the gut microbiota–SS interplay.

## 2. Materials and Methods

### 2.1. Study Design

**Figure 1:**
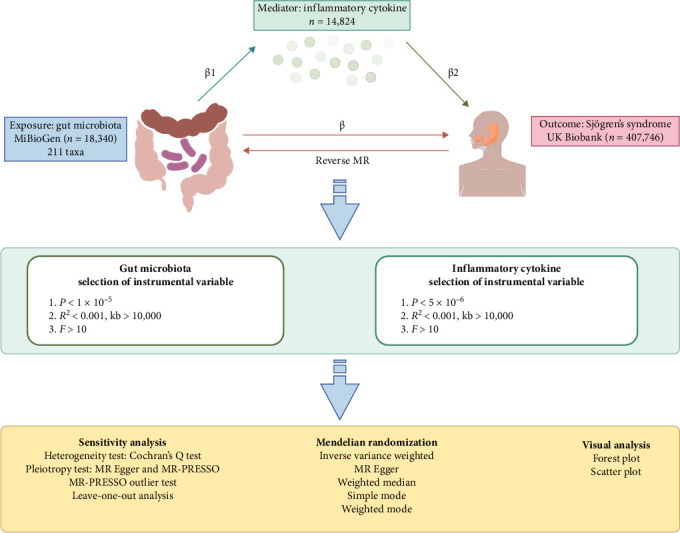
Study design and flow chart. ‘*β*' denotes the total effect of gut microbiota on SS, reverse MR illustrates the bidirectional causal relationship between the gut microbiota and SS ‘*β*1' indicates the MR effect of gut microbiota on mediators, and ‘*β*2' reflects the MR effect of mediators on SS, taking into account genetically determined gut microbiota. IVW, inverse-variance-weighted; MR, Mendelian randomization; MR-PRESSO, Mendelian Randomization Pleiotropy Residual Sum and Outlier. This figure was created by Figdraw (www.figdraw.com).

Figure 1 illustrates the overall analytical framework. This investigation leveraged summary statistics from existing GWAS to examine the genetic relationship between gut microbiota composition and SS. A multi-stage MR approach was employed to dissect the causal relationships and potential mediation mechanisms. Initially, bidirectional two-sample MR analytical approaches were employed to examine the causal effects of gut microbiota and inflammatory cytokines on SS risk, in respective terms. For associations that reached significance, we performed mediation analysis based on the two-step MR framework. Multivariable MR (MVMR) was subsequently used to quantify the direct effect of gut microbiota on SS, adjusting for the mediating effects of inflammatory cytokines. All estimations employed genetic variants—primarily single nucleotide polymorphisms (SNPs)—as IVs, in accordance with standard MR assumptions [20]. This research followed the Strengthening the Reporting of Observational Studies in Epidemiology Using MR (STROBE-MR) guidelines [21].

### 2.2. Data Sources

#### 2.2.1. Gut Microbiota Data Source

Summary-level data for gut microbiota were obtained from the MiBioGen consortium's genome-wide meta-analysis. This resource comprised 16S rRNA-derived microbial abundance profiles and genetic data from 18,340 individuals of predominantly European ancestry across 24 cohorts. Our analysis focused on 211 microbial taxa (ranging from phylum to genus level) for which genome-wide significant microbiome quantitative trait loci (QTLs) had been identified [22].

#### 2.2.2. Inflammatory Cytokine Data Source

Genetic associations for inflammatory cytokines were sourced from a GWAS meta-analysis, which measured 91 plasma protein levels using the Olink Target-96 Inflammation panel in 14,824 European-ancestry participants [23].

#### 2.2.3. SS Data Source

Summary statistics for SS were acquired from the UK Biobank [24], a large-scale prospective cohort. We utilized the European ancestry subset of this resource, which included 407,746 individuals of European ancestry after quality control and relatedness pruning. The analysis was based on imputed genotype data passing standard QC thresholds [25].

This investigation [26] constitutes a secondary analysis based on summary data from existing genome-wide association studies. Each original GWAS incorporated in the analysis had obtained prior ethical approval. It is important to emphasize that this analysis exclusively employed summary-level data, with no access to individual-level information, thereby precluding the need for additional ethical review board approval.

### 2.3. Instrumental Variable Selection

To ensure robust causal inference, genetic instruments for each exposure were selected according to standard MR principles. For gut microbiota taxa, a relaxed genome-wide significance threshold (*p* < 1 × 10^−5^) was adopted to identify associated SNPs, a common strategy to overcome the limited availability of instruments for microbial features [27–29]. For inflammatory cytokines, a threshold of *p* < 5 × 10^−6^ was applied to maximize the number of available instruments while maintaining stringency [29, 30]. Subsequently, all selected SNPs were clumped to ensure independence (linkage disequilibrium threshold *r*^2^ < 0.001 within a 10,000 kb window) using the 1000 Genomes European project as the reference panel [31]. Palindromic SNPs with ambiguous strand orientation were removed to prevent allele effect mismatching. Finally, the strength of each IV was quantified using the *F*-statistic, and all SNPs with an F-statistic greater than 10 were retained for analysis, mitigating concerns of weak instrument bias [32].

### 2.4. MR Analyses

#### 2.4.1. Two-Sample MR Analysis and Sensitivity Analysis

Causal effects of gut microbiota and inflammatory cytokines on SS were estimated employing two-sample MR. For exposures instrumented by multiple genetic variants, causal estimates were derived using a suite of methods: the inverse-variance weighted (IVW) approach served as the primary method due to its superior statistical power under the absence of horizontal pleiotropy [33]. To complement the IVW results and evaluate robustness, several additional methods were implemented: the weighted median method [34]; MR-Egger regression, which provides an estimate accounting for directional pleiotropy and allows its detection via the intercept test [35]; alongside the simple median and weighted mode methods as supplementary approaches [36].

Comprehensive sensitivity analyses were performed to assess result reliability. Heterogeneity across variant-specific estimates was evaluated using Cochran's Q statistic, with a random-effects IVW model applied when significant heterogeneity was detected (*p* < 0.05) [37]. Horizontal pleiotropy was examined via the MR-Egger intercept test and the MR-PRESSO framework, which also identifies and corrects for outliers [38, 39]. To control for multiple testing, the false discovery rate (FDR) was applied with a significance threshold of *q* < 0.1; associations with *p* < 0.05 but *q* ≥ 0.1 were considered suggestive. The influence of individual variants was evaluated using leave-one-out analysis. The overall causal effect was visualized using scatter plots, and forest plots were generated to display the estimates from each genetic instrument. Statistical power for the detected significant associations was calculated using an online tool (https://sb452.shinyapps.io/power).

### 2.5. Mediation Analysis

To explore potential mediating mechanisms, we conducted mediation analyses on gut microbiota taxa and inflammatory cytokines that showed robust causal associations with SS in our initial MR analyses. The analysis proceeded in two steps: first, we assessed the causal effect of the identified gut microbiota on the candidate cytokine mediators; second, for those exhibiting a significant association, we evaluated the causal effect of the cytokine on SS. The indirect (mediated) effect was then quantified by calculating the product of the two causal estimates (β1 × β2), where β1 denotes the effect of the gut microbiota on the cytokine and β2 represents the effect of the cytokine on SS. The proportion of the total effect mediated was subsequently derived by dividing this indirect effect by the total effect of the gut microbiota on SS. This approach aimed to determine the fraction of the gut microbiota's total causal effect on SS that operates through the inflammatory cytokine pathway. Confidence intervals (CIs) for the indirect effect were estimated using the delta method [40].

### 2.6. Reverse MR Analysis

To examine potential reverse causation, a bidirectional MR analysis was performed by conceptually reversing the exposure–outcome relationship: SS was set as the exposure, and gut microbiota taxa were analyzed as outcomes. IVs for SS were selected as SNPs reaching genome-wide significance (*p* < 5 × 10^−8^). Results are presented as odds ratios (ORs) with 95% CIs. A *p*-value threshold of less than 0.05 was used to indicate potential causal relationships.

All statistical analyses were conducted using R version 4.3.2 (http://www.Rproject.org). MR analyses were performed utilizing the R-based package “TwoSampleMR,” “RMediation,” and “MR PRESSO” [41].

## 3. Results

### 3.1. Effect of Gut Microbiota on SS

**Figure 2:**
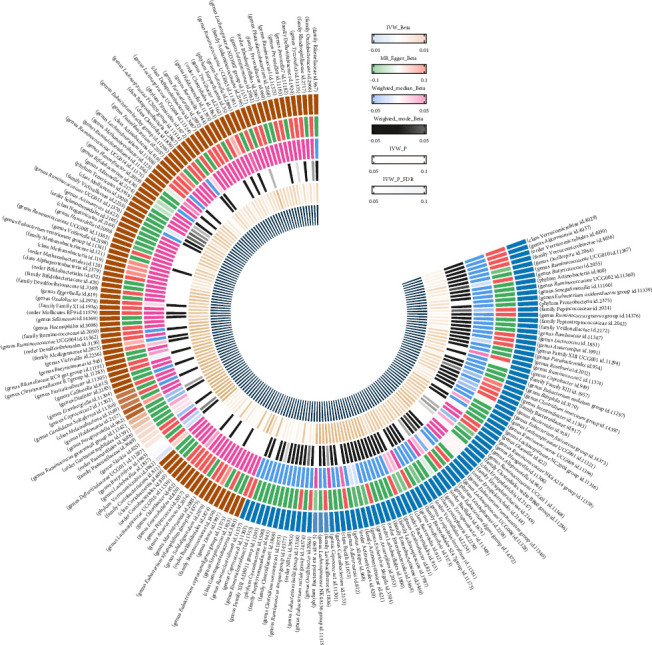
Shows the heat map of the causal relationship between gut microbiota and SS.

**Figure 3:**
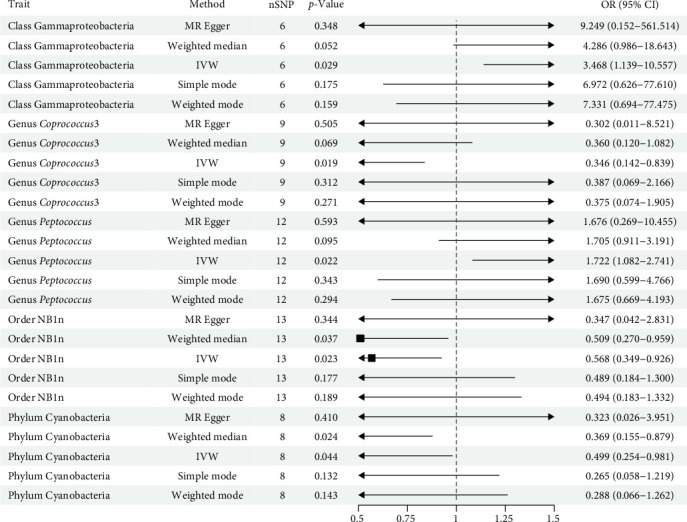
Mendelian randomization results of the causal relationship between gut microbiota and SS.

The analysis utilized 2559 SNPs as IVs for 193 gut microbiota traits (Supporting Information Table S1, Figure 2). After harmonization, a total of 2149 SNPs in the gut microbiota were retained for analysis, accounting for ~83.98% of the initially considered SNPs (Supporting Information Table S1-1). Among these, five gut microbiota traits (including one phylum, two genera, one order, and one class) showed associations with SS (Supporting Information Table S2, Figure 3). Detailed information on the 48 SNPs associated with these five gut microbiota traits is provided in Supporting Information Table S3. As depicted in Figure 3, MR analysis revealed that genetic predictions for two gut microbiota traits (class Gammaproteobacteria and genus Peptococcus) were associated with an increased risk of SS. Specifically, class Gammaproteobacteria (OR = 3.468, 95% CI = 1.139–10.557, *p*=0.029) and genus Peptococcus (OR = 1.722, 95% CI = 1.082–2.471, *p*=0.022) significantly elevated the risk of SS. Conversely, genetic predictions for three gut microbiota traits (genus *Coprococcus3*, order *NB1n*, and phylum Cyanobacteria) were linked to a decreased risk of SS. Genus *Coprococcus3* (OR = 0.356, 95% CI = 0.142–0.839, *p*=0.019), order *NB1n* (OR = 0.568, 95% CI = 0.349–0.926, *p*=0.023) and phylum Cyanobacteria (OR = 0.499, 95% CI = 0.254–0.981, *p*=0.044) significantly reduced the risk of SS. Nevertheless, these associations failed to maintain statistical significance following FDR adjustment for multiple testing. Heterogeneity tests for bacterial taxa and SS resulted in *p*-values above 0.05, suggesting no significant horizontal pleiotropy, which was consistent with MR-Egger findings. MR-PRESSO analysis also detected no outliers, strengthening confidence in the IVW results (Supporting Information Table S4). The reliability of the MR analyses was also supported by the results of “leave-one-out” analyses (Supporting Information Figure S1). A scatter plot summarizing the overall effect of gut microbiota on SS is provided in Supporting Information Figure S2, accompanied by a forest plot visualizing the causal relationship between gut microbiota and SS (Supporting Information Figure S3).

### 3.2. Effect of Inflammatory Cytokines on SS

**Figure 4:**
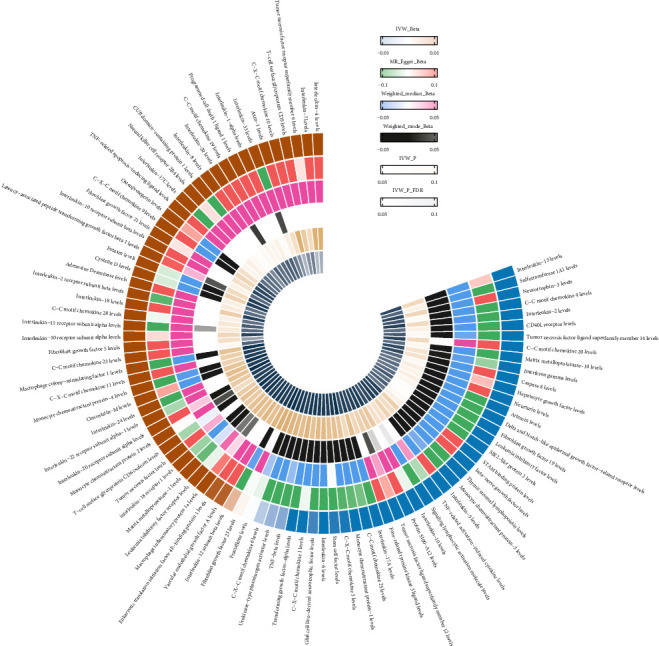
Shows the heat map of the causal relationship between inflammatory cytokines and SS.

**Figure 5:**
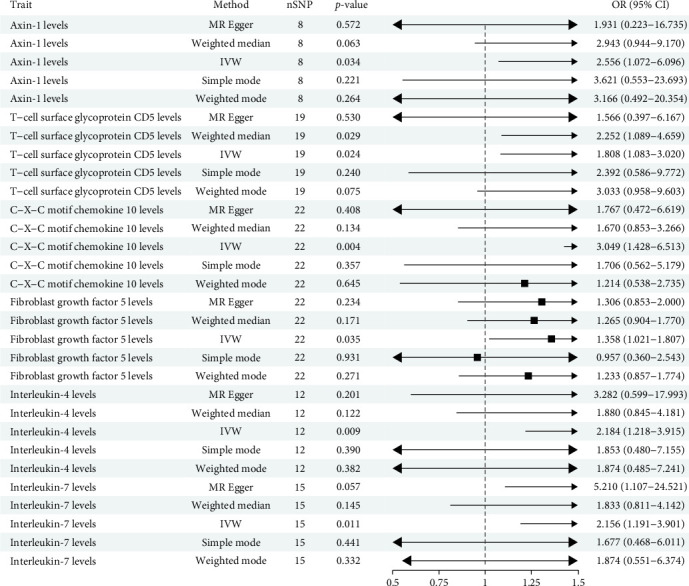
Mendelian randomization results for causal effects between cytokines and SS.

The analysis identified 1819 SNPs associated with 91 cytokines (Supporting Information Table S5, Figure 4). After harmonization, a total of 1379 SNPs in the inflammatory cytokine were retained for analysis, accounting for ~75.81% of the initially considered SNPs (Supporting Information Table S5-1). As depicted in Figure 5, a total of six inflammatory cytokines were found to be associated with SS. Details of the 98 SNPs for these cytokines are provided in Supporting Information Table S6. Elevated levels of C-X-C motif chemokine 10 (OR = 3.049, 95% CI = 1.428–6.513, *p*=0.004), Interleukin-4 (OR = 2.184, 95% CI = 1.218–3.915, *p*=0.009), Interleukin-7 (OR = 2.156, 95% CI = 1.191–3.901, *p*=0.011), T-cell surface glycoprotein CD5 (OR = 1.808, 95% CI = 1.083–3.020, *p*=0.024), Axin-1 (OR = 2.556, 95% CI = 1.072–6.096, *p*=0.034), and Fibroblast growth factor 5 (OR = 1.358, 95% CI = 1.021–1.807, *p*=0.035) were significantly associated with an increased incidence of SS, as detailed in Supporting Information Table S7. However, these associations did not retain statistical significance after applying FDR correction for multiple comparisons. No significant heterogeneity was observed between inflammatory cytokines and SS (all *p*  > 0.05), and MR-Egger regression detected no horizontal pleiotropy. The absence of outliers was further supported by MR-PRESSO, reinforcing the robustness of the IVW estimates (Supporting Information Table S4).

### 3.3. Effect of SS on Gut Microbiota

In the opposite pathway, SS showed associations with increased abundance of genus Dialister and genus Lachnospiraceae UCG001, as well as decreased abundance of genus *Ruminococcus gnavus* group. However, after FDR correction, associations with gut flora did not reach statistical significance (Supporting Information Table S8).

### 3.4. Mediation Analysis

We analyzed five gut microbiota taxa and six inflammatory factors, identifying significant associations through IVW analysis. Specifically, class Gammaproteobacteria showed a positive correlation with Axin-1 levels (OR = 1.300, 95% CI = 1.073−1.574, *p*=0.007), while genus Coprococcus3 was positively associated with C-X-C motif chemokine 10 levels (OR = 1.262, 95% CI = 1.056−1.508, *p*=0.010). After harmonization, a total of 48 SNPs in the gut microbiota were retained for analysis, accounting for ~80% of the initially considered SNPs. Using MVMR to identify inflammatory factors independently influencing SS after adjusting for gut microbiota effects, we quantified mediation effects and proportions. The analysis revealed that class Gammaproteobacteria exerted an indirect effect on SS through Axin-1 levels (β = 0.211, OR = 1.235, 95% CI = 1.01−1.66, *p*=0.027), accounting for 16.96% of the total effect, while genus Coprococcus3 mediated its effect through C-X-C motif chemokine 10 levels (β = 0.391, OR = 1.479, 95% CI = 1.081−2.232, *p*=0.001) with a mediation proportion of 36.78% (Table 1, Supporting Information Tables S9, S9-1).

## 4. Discussion

In this thorough MR study, suggestive evidence was found for potential causal links connecting five gut microbial taxa with SS. Through two-step MR and MVMR analyses, two mechanistic pathways were revealed, implicating two inflammatory cytokines and two microbial taxa that together demonstrate a partial mediating influence. Our findings suggest that class Gammaproteobacteria may increase the risk of SS by elevating Axin-1 levels, whereas genus Coprococcus3 appears to mediate SS risk via heightened C-X-C motif chemokine 10 expression. This work represents the first thorough investigation into the genetic associations between gut microbiota and SS, highlighting the fundamental involvement of gut microorganisms in host–microbiota interactions related to SS pathogenesis. These insights highlight the importance of balancing this microbiota–host interaction in both preventive and therapeutic strategies for SS.

Gut microbiota–host interactions are crucial for immune development, homeostasis, and maintaining gut barrier integrity. Emerging evidence indicates that gut microbiota play a regulatory role in multiple aspects of autoimmune diseases, including pathogenesis, therapeutic response, nutrient metabolism, and immune modulation. This dynamic relationship underscores the microbiota's significant role in autoimmune conditions, where dysbiosis may exacerbate symptoms or contribute to disease progression. Understanding these interactions offers insights into novel therapeutic strategies aimed at manipulating the microbiota to benefit autoimmune disease management [42–44]. The association between gut microbiota and the onset of SS has been investigated, with research showing an increased prevalence of opportunistic pathogenic bacteria. Conversely, beneficial or commensal butyric acid-producing bacteria are found in low levels. These changes may contribute to the chronic inflammatory process associated with pSS by increasing secretion of pro-inflammatory cytokines such as IL-6, IL-12, IL-17, and tumor necrosis factor-alpha. Additionally, they may downregulate levels of forkhead box protein 3 (Foxp3), crucial for regulatory T-cell (Treg) development and function, thereby impairing intestinal barrier function [18, 45].

Our MR study found that the overall protective effect of Coprococcus on SS is consistent with previous research. Coprococcus may suppress CXCL10 expression through SCFA-mediated anti-inflammatory effects, immunomodulation via tryptophan metabolites, and enhancement of intestinal barrier function, thereby influencing the pathogenesis of SS [46]. CXCL10, a Th1-type chemokine, binds to CXCR3 to promote the recruitment of immune cells such as T cells and monocytes. It is significantly elevated in various autoimmune diseases, including SS, exacerbating glandular inflammation and tissue damage [16]. Studies indicate that the enrichment of Coprococcus increases the production of butyrate and indole-3-propionic acid (IPA), where butyrate suppresses the NF-κB pathway [47] and IPA activates the AhR signaling pathway [48], collectively downregulating CXCL10 release. Additionally, Coprococcus may strengthen the intestinal barrier, reducing LPS translocation and indirectly inhibiting macrophage activation and CXCL10 secretion [49]. In SS, overexpression of CXCL10 promotes the infiltration of CXCR3+ Th1 cells into target organs (e.g., salivary glands), driving interferon-gamma (IFN-γ)-mediated autoimmune responses, while CXCL10 inhibition significantly alleviates glandular inflammation. Thus, Coprococcus may reduce CXCL10 levels through SCFA/IPA-dependent pathways, decreasing Th1 cell recruitment and IFN-γ production, thereby mitigating SS progression. The directional inconsistency in the mediation analysis may suggest the presence of a suppression effect. Observational studies might have captured the total effect rather than the independent role of the mediating pathway. This mechanistic insight advances our understanding of the gut microbiota–immune axis in SS and proposes the Coprococcus–CXCL10 pathway as a viable candidate for therapeutic development.

Gut microbiota dysbiosis may influence Axin-1 expression through multiple pathways, thereby contributing to the pathogenesis of SS. Current evidence suggests that microbial metabolites such as SCFAs can modulate Axin-1 expression [50], and as a key negative regulator of the Wnt/β-catenin signaling pathway, alterations in Axin-1 levels directly impact β-catenin stability [51]. Downregulation of Axin-1 induced by gut microbiota imbalance leads to reduced β-catenin degradation and consequent hyperactivation of Wnt signaling, which may promote Th1/Th17 cell differentiation and enhance production of pro-inflammatory cytokines including IFN-γ and interleukin-17, thereby exacerbating autoimmune-mediated inflammation in salivary glands [17, 52]. Importantly, genetic studies have identified associations between polymorphisms in Wnt/β-catenin pathway-related genes and SS susceptibility, providing indirect support for the involvement of the gut microbiota-Axin-1-Wnt/β-catenin axis in SS pathogenesis. While no direct evidence currently links specific microbial taxa such as Gammaproteobacteria with Axin-1 regulation, experimental studies have partially validated the general mechanism whereby gut microbiota influences Axin-1 expression through metabolic and immunological pathways. Our study represents the first demonstration of significant associations among gut microbiota, Axin-1, and SS, offering novel insights into the role of gut microbiota in autoimmune disorders and establishing a theoretical foundation for developing microbiota-targeted therapeutic strategies, although the precise regulatory mechanisms require further experimental elucidation.

Our mediation analysis offers genetic evidence supporting the association between gut microbiota and inflammatory cytokines, and to our knowledge, is the first to report a direct link between the class Gammaproteobacteria and Axin-1, as well as between Coprococcus3 and C-X-C motif chemokine 10. Although prior research has established a general relationship between gut microbiota and inflammatory cytokines, the specific regulatory influence of Gammaproteobacteria on Axin-1, as well as the direct association of Coprococcus3 with C-X-C motif chemokine 10, had not been previously demonstrated. This novel discovery significantly enhances our understanding of SS and could offer fresh insights into its early diagnosis and prevention. It has been reported that impairing intestinal barrier function promotes the chronic inflammatory process associated with SS. This impairment increases the secretion of pro-inflammatory cytokines such as IL-6, IL-12, IL-17, and tumor necrosis factor-alpha, while also downregulating the levels of Foxp3, which plays a critical role in the development and function of regulatory T cells (Tregs). Collectively, these results, in conjunction with our data, clarify the complex causal interplay involving gut microbiota, inflammatory cytokines, and the development of SS. However, given the complexity of the interactions between gut microbiota and SS, it is likely that other unmeasured mediators exist. Future studies could expand this investigation to include additional cytokines or immune pathways that might contribute to the mediation effect.

A key strength of MR in this investigation lies in its use of SNP as IVs to evaluate causal pathways between exposures and outcomes. Compared to randomized controlled trials (RCTs), MR minimizes bias from confounders and avoids interference from reverse causality. By analyzing publicly available GWAS datasets with large sample sizes, this study achieved more precise estimates. Crucially, the results remain robust against horizontal pleiotropy and other confounding factors.

However, several limitations warrant consideration. Firstly, the data are restricted to individuals of European descent, raising concerns about generalizability to broader populations. Secondly, potential overlap between exposures and outcomes among study participants poses a challenge; however, through the MR-Egger intercept test and sensitivity analysis, it is shown that these analysis results have no significant pleiotropic or heterogeneity and will not significantly affect our research results. Thirdly, our MR analysis did not adjust for gender, despite the known female predominance in SS (female-to-male ratio ~ 9:1). The lack of gender-stratified GWAS summary statistics in the UK Biobank limited our ability to explore potential sex-specific genetic effects. Given that sex hormones and immune responses are tightly linked, future studies should prioritize sex-stratified MR analyses to clarify whether causal relationships between gut microbiota, inflammatory cytokines, and SS differ by gender. Fourthly, our study relied on general population data from the UK Biobank, where SS patients may have comorbid autoimmune diseases. Although MR mitigates confounding by design, residual bias may persist if genetic variants associated with SS also influence microbiota or inflammation through overlapping pathways in other autoimmune conditions. Future studies with stratified SS cohorts are warranted to dissect these effects. Fifth, due to the limited case size, the ability to detect true causal effects was constrained, particularly for exposure–outcome associations with smaller effect sizes where sufficient statistical power may have been lacking, resulting in non-significant findings for some bacterial taxa, and thus the possibility of undetected true associations cannot be entirely ruled out; future studies could employ more explanatory polygenic scores, expand sample sizes, or integrate multi-cohort data to enhance statistical power, thereby improving the reliability of causal inference. Finally, challenges related to nonsignificance after multiple corrections underscore the importance of balancing biological plausibility with rigorous statistical approaches. Despite these limitations, the study holds academic significance, and future investigations should aim to validate these findings using diverse populations and larger sample sizes to enhance robustness.

Nevertheless, this study yields suggestive evidence for a potential causal pathway connecting gut microbiota, inflammatory cytokines, and SS. The genetic associations identified here may help direct future research into underlying mechanisms and clinical applications related to this autoimmune disorder.

## 5. Conclusion

In conclusion, our MR analysis revealed suggestive evidence for potential causal associations between five gut microbiota taxa and increased SS risk, while effectively ruling out reverse causation. Importantly, our findings indicate that Gammaproteobacteria influence SS development by elevating Axin-1 levels, while Coprococcus3 affects SS through C-X-C motif chemokine 10 expression. To deepen our understanding of the gut microbiota–SS link, future studies should explore potential mechanistic pathways. Although our findings contribute to understanding the gut microbiota–SS relationship, additional experimental and clinical studies are required to confirm and extend these results. We expect that our research will stimulate further inquiry in this area, ultimately contributing to ongoing efforts to improve patient outcomes in SS.

## Figures and Tables

**Table 1:** Two-step Mendelian randomization.

Exposure	Outcome	Mediation	Total effect (*β*)	A (*β*)	B (*β*)	Indirect effect (*β*)	Mediation effect/total effect	95% CI
Class Gammaproteobacteria	Sjogren's syndrome	Axin-1 levels	1.244	0.262	0.805	0.211	16.96%	1.01–1.66
Genus Coprococcus3	Sjogren's syndrome	C-X-C motif chemokine 10 levels	−1.063	0.233	1.681	0.391	36.78%	1.081–2.232

*Note:* A = the influence of the exposure on the mediator. B = the association between the mediator and the outcome remains significant after adjusting for the exposure.

## Data Availability

All GWAS summary data are publicly available. Summary statistic data for GM at the genus level were collected from MiBioGen (https://mibiogen.gcc.rug.nl/), which included 119 genera (ebi-a-GCST90016908 to ebi-a-GCST90017118). The GWAS data sets for 91 inflammatory factors are available in the GWAS Catalog (https://www.ebi.ac.uk/gwas/ accession numbers from GCST90274758 to GCST90274848). The genetic data of SS are available at IEU Open GWAS (https://gwas.mrcieu.ac.uk/datasets/ebi-a-GCST90013879/).

## Supporting Information

Supporting Information 1 Figures S1-S3: The plots of MR analysis results.

Supporting Information 2 Table S1: 2559 SNPs for the 193 gut microbiota taxa. Table S1-1: Harmonized data of Gut Microbiota and Sjögren's Syndrome.

Supporting Information 3 Table S2: The causal effects of gut microbiota on SS.

Supporting Information 4 Table S3: The characteristics of 48 SNPs analyzing the causal effects of the 5 gut microbiota on SS.

Supporting Information 5 Table S4: Mendelian randomization sensitivity analysis of SS.

Supporting Information 6 Table S5: 1819 SNPs for the 91 cytokines. Table S5-1: Harmonized data of cytokines and Sjögren's Syndrome.

Supporting Information 7 Table S6: The characteristics of 98 SNPs analyzing the causal effects of the 6 cytokines on SS.

Supporting Information 8 Table S7: The causal effects of cytokines on SS.

Supporting Information 9 Table S8: The causal effects of SS on gut microbiota.

Supporting Information 10 Table S9: Causal effects of gut microbiota associated with SS on cytokines associated with SS. Table S9-1: Harmonized data of gut microbiota and inflammatory cytokine.
