# Supplementary material for: The Role of Inflammatory Cytokines in the Causal Pathway From Gut Microbiota to Sjögren's Syndrome: Evidence From Mendelian Randomization
Source: Mediators Inflamm. 2025 Dec 1;2025:1951493. doi: 10.1155/mi/1951493 (PMC12685423; doi:10.1155/mi/1951493)
Supplement: Supporting Information 1 — Figures S1-S3: The plots of MR analysis results. [file 1951493.f1.docx]

**Supplementary Figs**

**Fig S1.** MR leave-one-out sensitivity analysis for Gut microbiota on SS.

**Fig S2.** Scatter plots for the effect of Gut microbiota on SS.

**Fig S3.** Forest plots for the effect of Gut microbiota on SS.

**Fig S1.** MR leave-one-out sensitivity analysis for Gut microbiota on SS


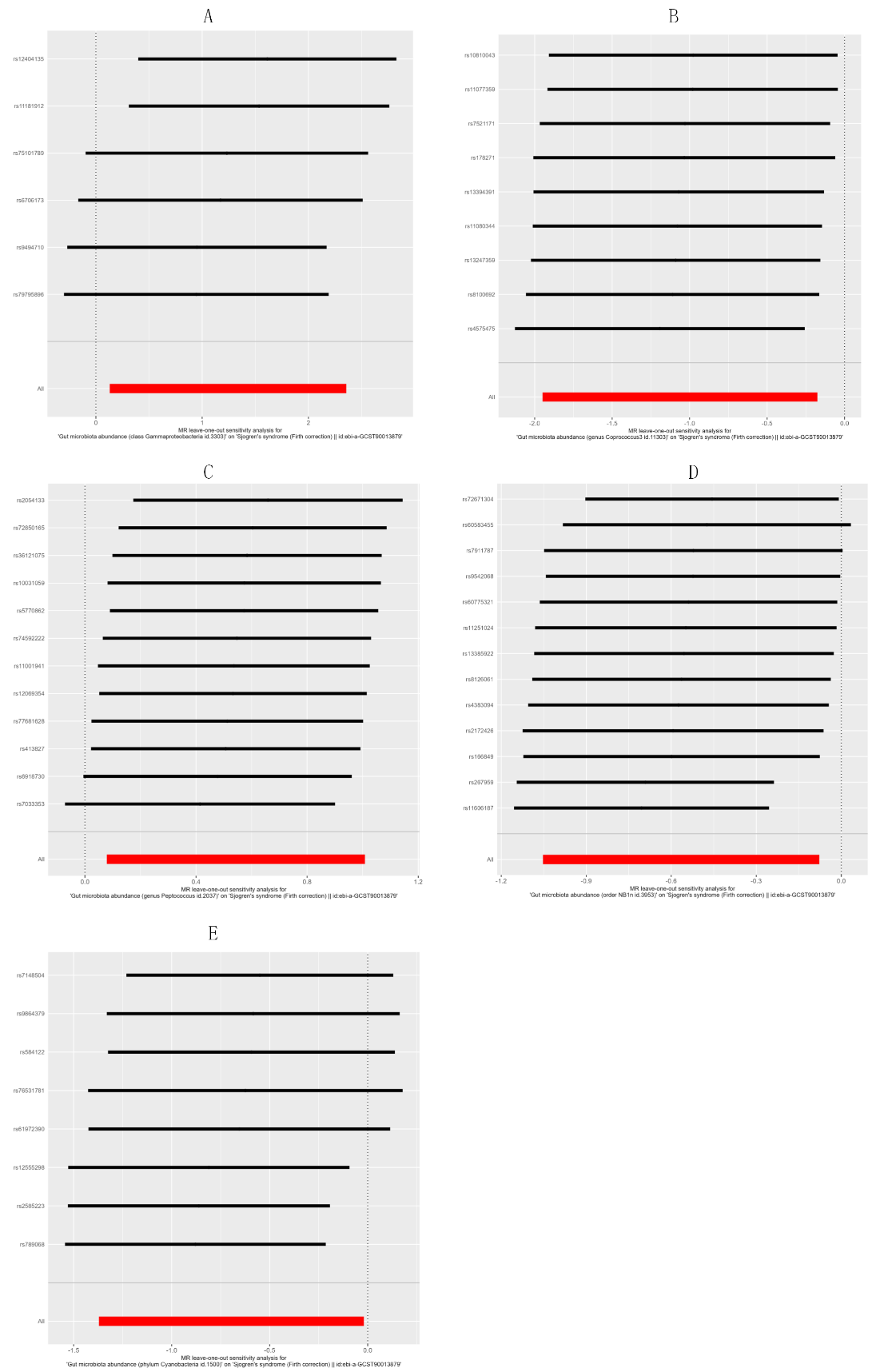


1. Analysis for "class Gammaproteobacteria id.3303" on "SS"

(B) Analysis for "genus Coprococcus3 id.11303" on "SS"

(C) Analysis for "genus Peptococcus id.2037" on "SS"

(D) Analysis for "order NB1n id.3953" on "SS"

(E) Analysis for "phylum Cyanobacteria id.1500" on "SS"

**Fig S2.** Scatter plots for the effect of Gut microbiota on SS


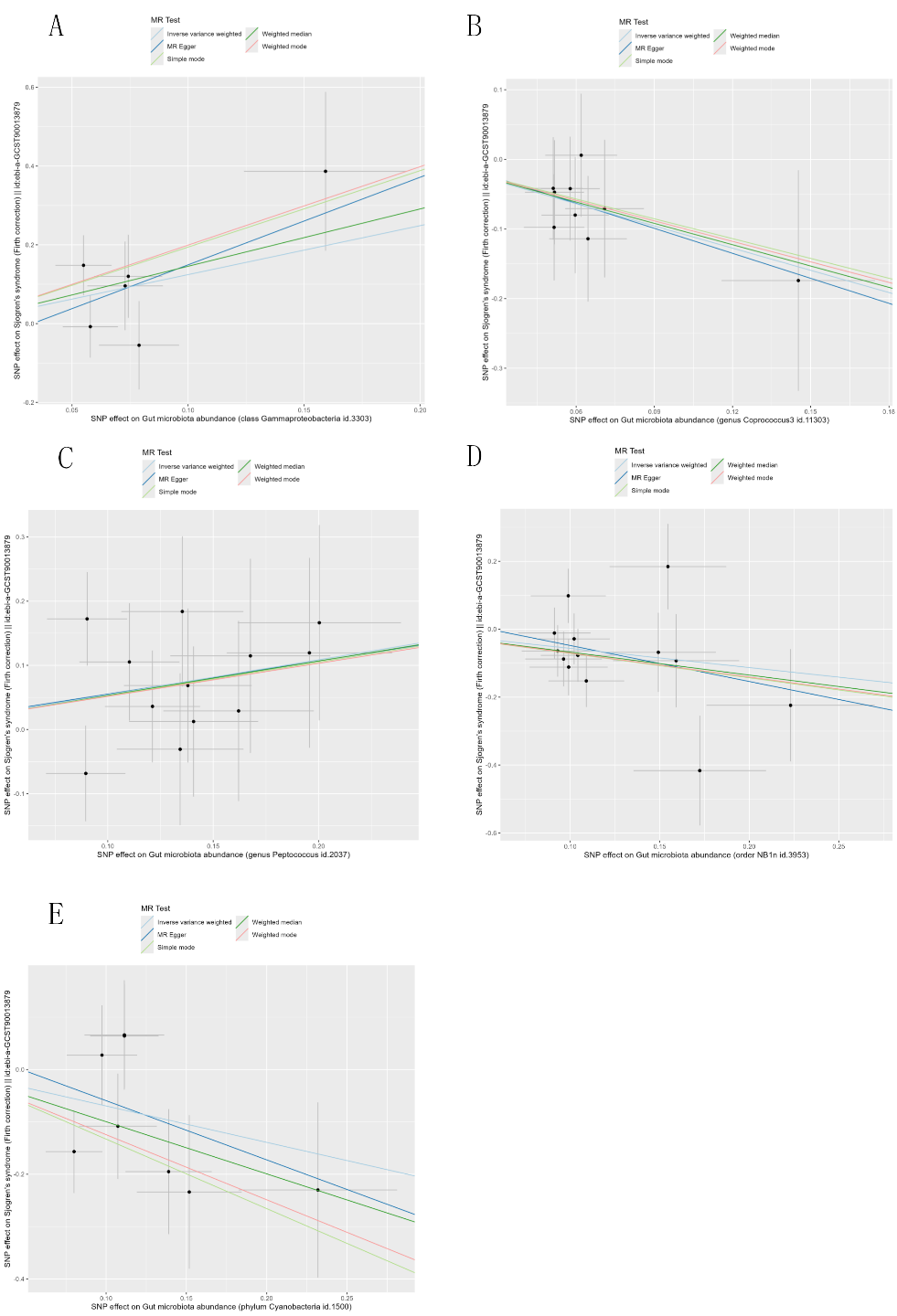


(A) MR effect size for "class Gammaproteobacteria id.3303" on "SS"

(B) MR effect size for "genus Coprococcus3 id.11303" on "SS"

(C) MR effect size for "genus Peptococcus id.2037" on "SS"

(D) MR effect size for "order NB1n id.3953" on "SS"

(E) MR effect size for "phylum Cyanobacteria id.1500" on "SS"

**Fig S3.** Forest plots for the effect of Gut microbiota on SS.


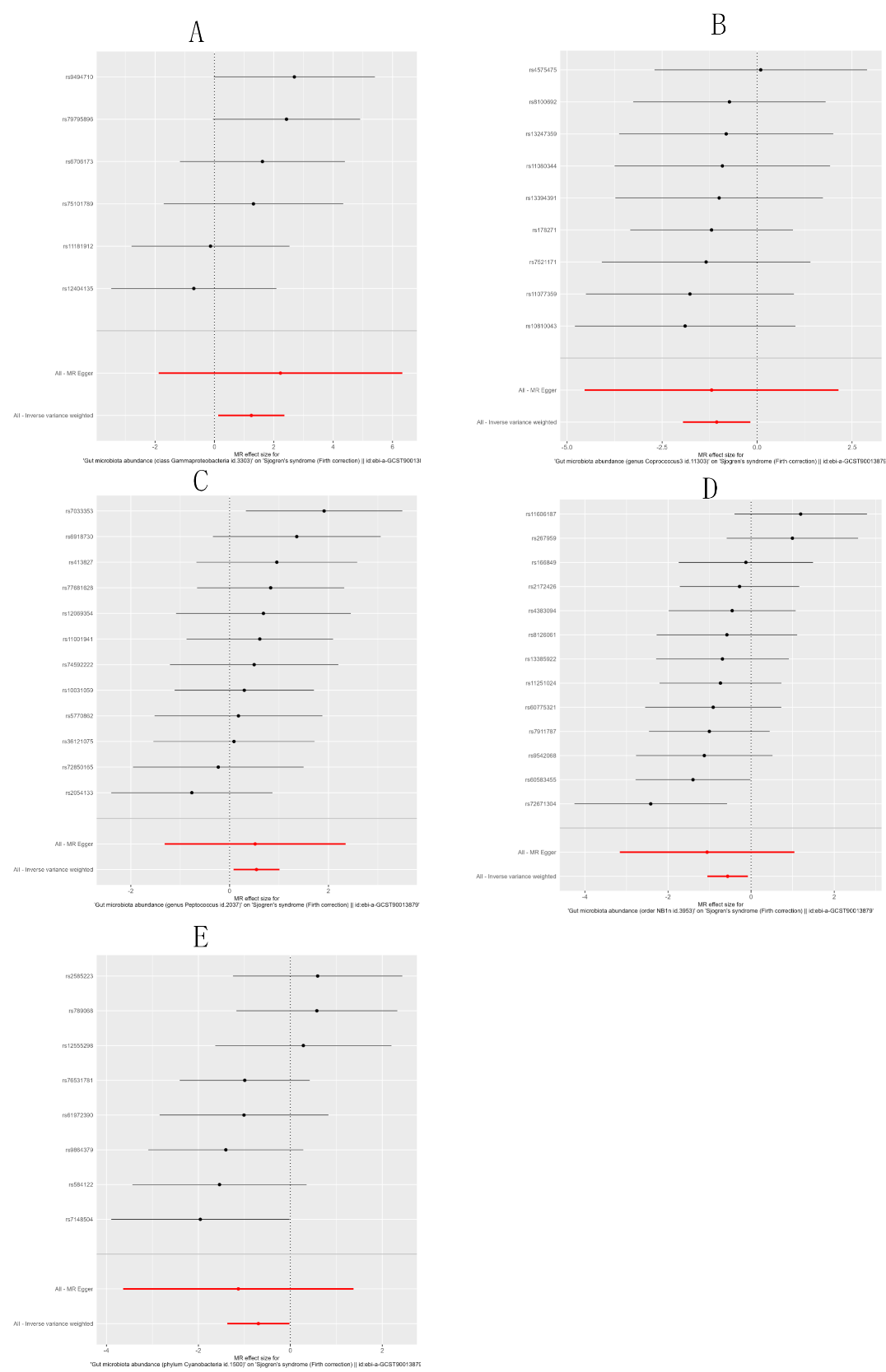


(A) MR effect size for "class Gammaproteobacteria id.3303" on "SS"

(B) MR effect size for "genus Coprococcus3 id.11303" on "SS"

(C) MR effect size for "genus Peptococcus id.2037" on "SS"

(D) MR effect size for "order NB1n id.3953" on "SS"

(E) MR effect size for "phylum Cyanobacteria id.1500" on "SS"
